# The Effectiveness of Additional Information Provided Before Colonoscopy Regarding Bowel Preparation

**DOI:** 10.3390/healthcare13040400

**Published:** 2025-02-13

**Authors:** Mehmet Sait Berhuni, Hüseyin Yönder, Hasan Elkan, Baran Yüksekyayla, Ali Uzunköy

**Affiliations:** Faculty of Medicine, Department of General Surgery, Harran University, Sanliurfa 63200, Türkiye; hyonder@hotmail.com (H.Y.); dr_elkan@hotmail.com (H.E.); brn_yuksekyayla@hotmail.com (B.Y.); aliuzunkoy@yahoo.com (A.U.)

**Keywords:** additional information, bowel preparation, colonoscopy

## Abstract

**Background:** This study aimed to evaluate the effect of patient education provided through one-on-one verbal instruction supported by visual media tools for bowel preparation prior to a colonoscopy procedure. **Materials and Methods:** This prospective study included patients who underwent colonoscopy in our clinic’s endoscopy unit between April 2024 and August 2024. The study design included two groups: the control group (CG), wherein patients scheduled for a colonoscopy received standard information about the procedure at the outpatient clinic, and the information group (IG), wherein patients were invited to the clinic 5 days before the procedure and received an interactive education session lasting approximately 10 min in addition to the standard information. The adequacy of bowel preparation was evaluated using the Boston Bowel Preparation Scale (BBPS). **Results:** This study included 228 patients, with 114 patients in each group. Of the patients, 137 were male (60.08%) and 91 were female (39.92%). The mean age of the patients was 49.41 ± 15.04 years, the mean BMI was 27.48 ± 4.23, and the mean BBPS score was 7.38 ± 1.96. The mean BBPS score of the patients in the IG and CG was 7.76 ± 1.84 and 7.00 ± 2.01, respectively, and there was a statistically significant difference between the two groups (*p* = 0.003). **Conclusions:** Additional information provided prior to the colonoscopy procedure was identified as an effective parameter on adequate bowel preparation.

## 1. Introduction

Colonoscopy is widely used today, primarily for the screening and diagnosis of colorectal cancers, as well as in the management of various other diseases [1,2]. The use of colonoscopy as a screening method in colorectal cancer prevention programs and as a tool for diagnosis, treatment, and follow-up in various conditions has led to an increase in the frequency of colonoscopies in many countries. Effective colonoscopy relies heavily on the cleanliness of the colon or the quality of bowel preparation [3]. Despite the importance of bowel cleanliness, suboptimal bowel preparation can occur in up to 25% of colonoscopy procedures [4]. Poor bowel preparation can lead to decreased cecal intubation rates, the reduced detection of intraluminal pathologies, prolonged colonoscopy times, increased patient discomfort, and higher costs due to the need for early repeat procedures [5,6].

Several scales are available for assessing the quality of bowel preparation [4]. The Boston Bowel Preparation Scale (BBPS) is a reliable scoring system used in clinical practice to evaluate bowel preparation [7]. One advantage of the BBPS is that scoring is conducted after the colon lumen has been washed and aspirated. Higher scores indicate good and adequate bowel preparation. In a recent study by Kim et al., cases with a total BBPS score of 6 were compared to those with a total score in the range of 7–9, and a significant decrease in the detection of luminal lesions was found in cases with a score of 6 [8].

Studies have indicated that inadequate bowel preparation is significantly more prevalent in populations with low levels of education and health literacy [2,4]. Consequently, it has been suggested that educational models utilizing various media tools could be beneficial in ensuring adequate bowel preparation [9]. Elvas et al. found that personalized patient education is significantly associated with better bowel preparation for colonoscopy [10].

This study aimed to evaluate the impact of education, reinforced with visual media tools and delivered through one-on-one verbal instruction, on bowel preparation in patients undergoing colonoscopy using BBPS.

## 2. Materials and Methods

### 2.1. Study Design and Patient Population

**Figure 1:**
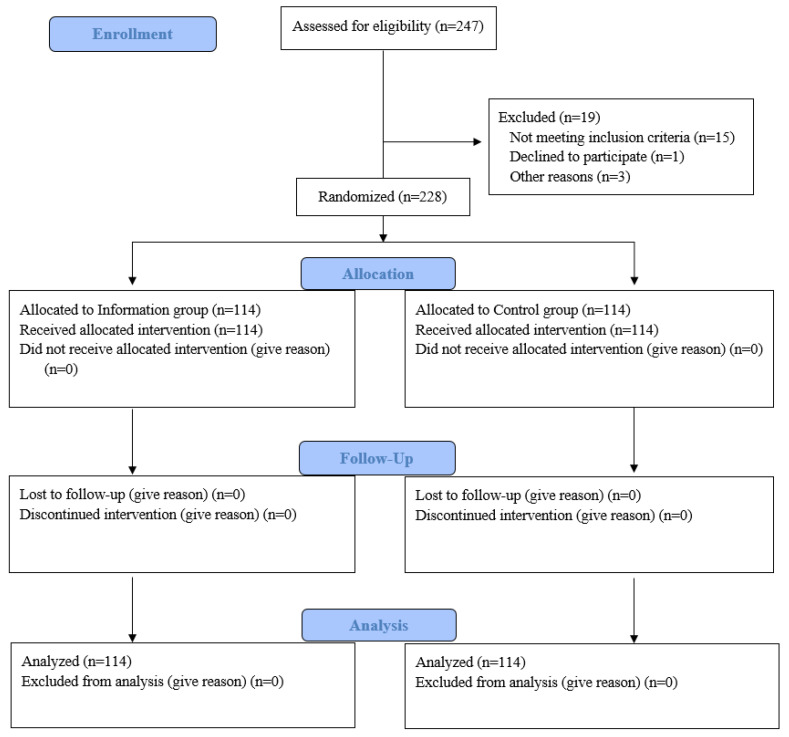
Patient selection flow chart.

This prospective study included 228 patients who underwent colonoscopy between April 2024 and August 2024. The study was designed by creating two groups through randomization achieved by selecting the first patient via drawing lots and then assigning subsequent patients to the groups sequentially (Figure 1). The study was conducted in adherence to the principles of the Declaration of Helsinki. Informed consent was obtained from each patient included in the study. Ethical approval was obtained from the institutional committee (date: 4 January 2024; decision number: 24.03.41).

### 2.2. Exclusion Criteria

Patients under 18 years of age, pregnant women, patients with a history of abdominal surgery, patients with an intellectual disability, those unwilling to participate in the study, patients who had previously undergone colonoscopy and who had experience of the procedure, patients for whom sufficient time was not available to follow the pre-procedure diet, those who were unable to correctly use the prescribed laxative solutions, and those requiring emergency procedures were excluded from the study.

### 2.3. Preparation Before Colonoscopy

In the control group (CG), patients received standard information from the outpatient clinic and were given a brochure containing explanatory details about the procedure. In the information group (IG), in addition to the measures taken for the CG, patients were scheduled for an appointment 5 days before the procedure. During this appointment, they received an approximately 10 min interactive presentation about the colonoscopy procedure and bowel preparation. This presentation, which utilized videos and visual aids, included one-on-one verbal instruction of the patient, followed by a session where the patient’s concerns were addressed and any additional questions were answered.

In both groups, a clear liquid diet was recommended starting 2 days before the procedure to ensure colon cleanliness. In all patients included in the study, bowel preparation was performed using two doses of 45 mL of oral sodium phosphate (sodium phosphate 45 mL, Recordati Pharmaceuticals, Milan, Italy) at 7:00 PM and 9:00 PM on the day before the procedure. On the morning of the procedure, all patients additionally received a single dose of a rectal enema (sodium phosphate 210 mL, Avixa Pharmaceuticals, Istanbul, Turkey) at 8:00 AM. All patients underwent colonoscopy under sedation provided by the anesthesia team and colonoscopy procedures were performed by two general surgeons who were blinded to the patient groups.

### 2.4. BBPS

**Figure 2:**
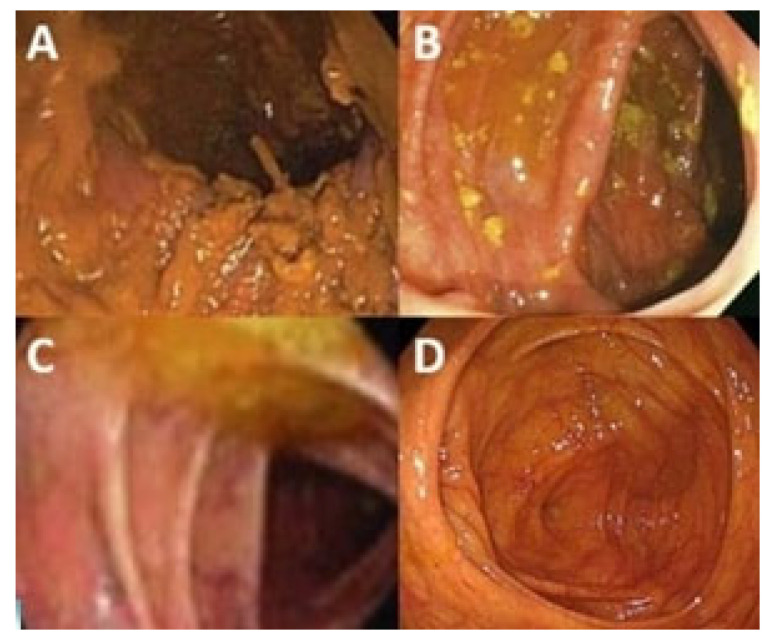
BBPS scoring during colonoscopy: (**A**) 0 points; (**B**) 1 point; (**C**) 2 points; (**D**) 3 points.

The BBPS, which has been widely accepted and validated for this purpose since 2009 [7], was used to evaluate the adequacy of bowel preparation. Based on this scale, after irrigation and aspiration of the ascending, transverse, and descending colon, each was assessed with a score from 0 to 3. A score of 0 was given if the colon mucosa was not visible due to solid stool and cleaning could not be achieved through irrigation. A score of 1 was given if part of the mucosa was visible, but the rest was obscured by debris, residual stool, or stool that was liquid but not clear. A score of 2 was given if there was minor contamination with stool, small fragments, and/or liquid but not clear stool, yet the mucosa of the colon segment was adequately visible. A score of 3 was given if the mucosa of the colon segment was completely clean and fully visible without any stool. The total scores for the three segments were summed, resulting in a final score ranging from 0 to 9 (Figure 2).

The patients’ age, sex, body mass index (BMI), education level, presence of chronic diseases, use of chronic medications, whether they received information, and BBPS scores were recorded. In this study, diabetes and hypertension, which are known to reduce bowel motility and are commonly observed in the population, were analyzed as chronic diseases. Therefore, the term chronic medication use was used to describe cases of patients who were on continuous medication due to these two conditions.

## 3. Statistical Analysis

Statistical analyses were performed using IBM SPSS Statistics for Windows, Version 25.0 (IBM Corp., Armonk, NY, USA). The Shapiro–Wilk test was utilized to assess the normality of the data distribution. Descriptive statistics for numerical variables were expressed as means ± standard deviations. Due to the non-normal distribution of the data, comparisons between the two groups were conducted using the Mann–Whitney U test. All analyses were executed with a 95% confidence interval, and statistical significance was defined by a two-tailed *p*-value of <0.05.

### Sample Size Determination

Based on the BBPS scores of all patients, a post hoc power analysis was conducted at a 0.05 significance level with a total sample size of 228 (*n*_1_ = 114, *n*_2_ = 114), yielding an effect size of 0.78 and a power of 0.99.

## 4. Results

Of the 228 patients included in this study, 137 were male (60.08%) and 91 were female (39.92%). The mean age of the patients was 49.41 ± 15.04 years, the mean BMI was 27.48 ± 4.23, and the mean BBPS score was 7.38 ± 1.96. There were no significant differences between the IG and CG in terms of age, gender, and BMI distribution (*p* > 0.05). The mean BBPS score of the 114 patients in the IG and 114 patients in the CG were 7.76 ± 1.84 and 7.00 ± 2.01, respectively, and there was a statistically significant difference between the two groups (*p* = 0.003). The number of patients with a BBPS of 5 or less, indicating inadequate bowel preparation, was 9 (7.9%) in the IG, compared to 20 (17.5%) in the CG, and a significant difference was observed between the groups (*p* = 0.029).

During colonoscopy, polyps were detected in 31 patients (27.2%) in the IG, while this number was 21 (18.4%) in the CG. There were no significant differences between the groups in this regard (*p* = 0.114). The total of 52 polyps detected were histopathologically evaluated, revealing 19 cases (36.5%) of hyperplastic polyps, 29 cases (55.8%) of adenomas (22 tubular, 5 tubulovillous, 2 villous), and 4 cases (7.7%) of high-grade dysplasia. In the IG, three patients (2.63%) had adenocarcinoma, while one patient (0.87%) in the CG had adenocarcinoma.

When the patients were evaluated according to sex, the mean BBPS score was 7.27 ± 2.01 in female patients and 7.56 ± 1.89 in male patients. Although the mean BBPS score was higher in female patients, no statistically significant differences were observed (*p* = 0.263).

When patients were divided into two groups based on age, no significant differences were found in BBPS scores between those ˃50 years of age and those aged < 50 (*p* = 0.609). There were also no significant differences in BMI between the two age groups (*p* > 0.05). Among the 122 patients < 50 years of age, 67 were in the IG and 55 were in the CG. For patients < 50 years of age, the mean BPPS score was 8.02 ± 1.48 in the IG and 6.74 ± 2.11 in the CG. A statistically significant difference was observed between these two groups (*p* < 0.05). Among the 106 patients aged ≥ 50, 47 were in the IG with a mean BBPS score of 7.38 ± 2.23, and the remaining 59 patients were in the CG with a mean BBPS score of 7.25 ± 1.88. No significant differences were found between these two groups (*p* > 0.05).

The patients were also evaluated based on BMI (those with a BMI ≥ 30 vs. those with a BMI < 30), and no significant differences were found in terms of age and sex distribution between the two groups (*p* > 0.05). Among the 175 patients with a BMI < 30, the mean BBPS score was 7.46 ± 1.88. The mean BBPS score was 7.11 ± 2.20 among the 53 patients with a BMI ≥ 30, and no statistically significant differences were observed between these two groups (*p* = 0.249). Among the 175 patients with BMI < 30, 89 were in the IG and 86 were in the CG. The mean BBPS score was 7.85 ± 1.68 in the IG and 7.06 ± 1.99 in the CG, with a statistically significant difference between the two groups (*p* = 0.006). Of the 53 patients with a BMI ≥ 30, 25 were in the IG and 28 were in the CG. Their mean BBPS scores were 7.44 ± 2.36 and 6.82 ± 2.05, respectively, and no significant differences were found between these two groups (*p* = 0.310).

Of the 72 patients with chronic diseases, 36 were in the IG and the remaining 36 were in the CG. For patients with chronic diseases, the mean BPPS score was 7.69 ± 1.16 in the IG and 7.05 ± 2.01 in the CG, and no statistically significant differences were found between these two groups (*p* = 0.104). Among the 156 patients without chronic diseases, 78 were in the IG and 78 in the CG. The mean BBPS score was 7.79 ± 2.09 in the IG and 6.98 ± 2.01 in the CG, with a statistically significant difference between these two groups (*p* = 0.015). The same situation was observed with regard to chronic medication use. Among the 65 patients with a history of chronic medication use, 35 were in the IG and 30 in the CG. No significant differences in BBPS scores were found between these patients (*p* = 0.37). However, among the 163 patients without a history of chronic medication use, 79 were in the IG and 84 in the CG, with a statistically significant difference in mean BBPS scores between these two groups (*p* = 0.006) (Table 1).

**Figure 3:**
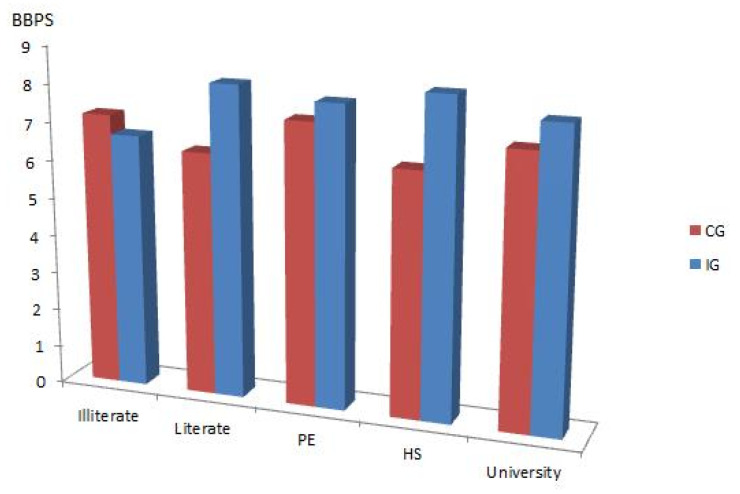
The relationship between education level and Boston Bowel Preparation Scale scores. (IG: information group; CG: control group; BBPS: Boston Bowel Preparation Scale; PE: primary education; HS: high school).

When examining the effects of educational level on bowel cleanliness, a statistically significant difference was found between the IG and CG for the literate and high school group (*p* < 0.05). The relationship between educational level and BBPS scores is shown in Figure 3.

## 5. Discussion

Inadequate bowel preparation can lead to numerous problems that impact the effectiveness of colonoscopy [5,8]. These issues include reduced cecal intubation rates, decreased detection rates of adenomas/polyps in the lumen, prolonged colonoscopy durations, increased patient discomfort, and higher costs due to early repeat procedures [4,11,12]. Studies have shown a significant difference in adenoma detection rates between patients with insufficient bowel preparation (total BBPS < 6) and those with adequate bowel preparation (total BBPS 6–7) [11,12]. However, some studies suggest that this relationship is not linear. For instance, in the study by Rai et al., no significant difference in adenoma detection rates was found between groups with good bowel preparation (total BBPS 6–7) and excellent bowel preparation (total BBPS 8–9) [13]. Risk factors for inadequate bowel preparation include advanced age, male sex, obesity, constipation, hypertension, diabetes, cirrhosis, stroke, the use of tricyclic antidepressants, a history of abdominopelvic surgery, long appointment times, low socioeconomic status, limited health literacy, low patient education levels, and insufficient pre-procedure patient education. Additionally, the effectiveness, amount, and duration of use of bowel preparation solutions can also play a role in inadequate bowel preparation [9,14,15].

In our study, the rate of inadequate bowel preparation in the control group was found to be 17.5%. In a multicenter study conducted by Fuccio et al. in Italy, which included 1524 patients, the rate of inadequate bowel preparation was reported to be 32% [16]. A study from France reported this rate as 25% [17]. A more recent study from China also reported a rate of 25.1% [18]. These differing rates obtained from various regions around the world are likely due to differences in socioeconomic status, dietary habits, and the designs of the studies.

Studies on various diseases have shown that patient education utilizing certain media tools can provide targeted benefits [19,20,21,22]. There are also studies indicating that education enhanced with visual media tools improves patient satisfaction and is effective in achieving appropriate bowel preparation for colonoscopy [23,24]. Spiegel et al. found that patients who received a 10 min video education in a Question and Answer format had significantly better bowel preparation than the CG [25]. In a recent study by Pattarapuntakul et al., it was reported that the study group who were prepared for colonoscopy through a smartphone application with video animations and a Question and Answer section and who were given a contact number to obtain further tinformation before the procedure if needed had statistically significantly higher BBPS scores compared to the CG [26]. In the present study, we also found that timely patient education close to the procedure had a significant positive effect on bowel preparation. This effect may be due to patients who received standard information from the outpatient clinic not adequately following diet and medication instructions, likely because they are provided too far in advance of the procedure. As the time between the standard information being provided to the patient and the procedure lengthens, the likelihood that patients will clearly remember the preparation process decreases.

In the study by Walton et al., advanced age and higher BMI were found to be significantly associated with lower BBPS scores [27]. This significant relationship is also supported by Feng et al.’s meta-analysis, as well as the study by Zhang et al. [2,28]. In these studies, an age of 60 years and a BMI threshold of 25 kg/m^2^ were accepted as the cut-off values. However, in the present study, the cut-off values were set at 50 years of age and a BMI of 30 kg/m^2^. Patients over the age of 50 in the present study had lower BBPS scores compared to those aged < 50, but this difference was not statistically significant. Although no significant difference was observed in BBPS scores between patients ˃ 50 and <50 years of age, it was found that providing information had no effect on BPPS scores in patients ˃ 50 years of age, whereas it made a significant difference in patients < 50 years of age. In the present study, patients with a BMI ≥ 30 had lower BBPS scores than those with a BMI < 30, although the difference was not statistically significant. However, while the information provided did not create a significant difference in BBPS scores among patients with a BMI ≥ 30, those with a BMI < 30 who received the information had significantly higher BBPS scores compared to those who did not receive the information. These findings suggest that as age and BMI increase, the positive contribution of the information provided to bowel preparation diminishes.

In their meta-analysis, Feng et al. indicated that apart from advanced age and BMI, male gender, the presence of comorbidities, chronic medication use, and education level are moderately associated with inadequate bowel preparation [2]. This meta-analysis is a valuable study in that it presents data from 125 studies, including 91 observational studies and 34 randomized controlled trials, published worldwide on this topic. Zhang et al. also identified male gender and the presence of comorbidities, in addition to advanced age and BMI, as risk factors for inadequate bowel preparation [28]. In the present study, although male gender was associated with lower BBPS scores, there were no significant differences compared to female patients. The presence of comorbidities and chronic medication use did not result in a significant difference in BBPS scores. However, while there were no significant differences in BBPS scores between the informed and uninformed groups in patients with comorbidities or a history of chronic medication use, informed patients without comorbidities or a history of chronic medication use had significantly higher BBPS scores compared to the uninformed group. This indicates that chronic disease and chronic medication use weaken the effectiveness of pre-procedural information on bowel cleanliness in colonoscopy patients.

In the study conducted by Ray et al., it was found that as patients’ education levels decreased, the rates of inadequate bowel preparation significantly increased [9]. In the present study, instead of evaluating patients based solely on education level, we assessed the effectiveness of information provided to patient groups with different education levels. In the informed group, patients who were literate but had no formal education and high school graduates had significantly higher BBPS scores, whereas no significant differences were observed in the informed group among illiterate patients, primary school graduates, and university graduates.

There are certain limitations to the present study. The first is that it was conducted at a single center. Another limitation is the relatively small sample size, which may affect the generalizability of the results. Additionally, this study did not assess the indications for colonoscopy, and different indications (e.g., chronic constipation) might influence bowel motility and preparation differently. Furthermore, individual differences in how patients perceive the verbal information provided could affect its effectiveness. These perceptual differences should be taken into account when interpreting the results.

In conclusion, providing comprehensive information several days before a colonoscopy was identified as a significant parameter for ensuring adequate bowel preparation. For an effective colonoscopic examination, it is crucial for patients to receive thorough education as close to the procedure as possible for optimal bowel preparation. Our findings may assist clinicians in identifying individuals at high risk for inadequate bowel preparation, and future, larger-scale studies could support these findings, providing broader insights for clinical practice. It would be beneficial to validate these results with studies involving larger patient cohorts.

## Figures and Tables

**Table 1:** Effect of additional information on demographic data and some parameters.

	Total	Groups	BBPS ^α^	*p* Value ^¥^	Total	Groups
		IG	CG	IG	CG	
All Patients	228	114	114	9.00 (2.00)	7.00 (3.00)	*p* = 0.003 *
Age						
<50	122	67	55	9.00 (1.75)	7.00 (3.00)	*p* = 0.001 *
>50	106	47	59	8.00 (3.00)	8.00 (3.00)	*p* = 0.748
Gender						
Female	91	48	43	9.00 (1.75)	8.00 (3.00)	*p* = 0.009 *
Male	137	66	71	8.50 (2.00)	7.00 (3.00)	*p* = 0.016 *
BMI						
<30	175	89	86	9.00 (2.00)	7.50 (3.00)	*p* = 0.006 *
>30	53	25	28	8.00 (3.00)	7.50 (3.00)	*p* = 0.310
Chronic Disease						
Yes	72	36	36	7.50 (3.00)	8.00 (3.00)	*p* = 0.104
No	156	78	78	8.50 (2.00)	7.00 (3.00)	*p* = 0.015 *
Chronic Drug Use						
Yes	65	35	30	8.00 (3.00)	7.50 (3.00)	*p* = 0.372
No	163	79	84	9.00 (2.00)	7.50 (3.00)	*p* = 0.006 *

IG: information group; CG: control group; BMI: body mass index; BBPS: Boston Bowel Preparation Scale. ^α^ Median (interquartile range), ^¥^ Mann–Whitney U test, * *p* in bold indicates statistical significance.

## Data Availability

Data are contained within the article.
